# Deep-sequencing of Marburg virus genome during sequential mouse passaging and cell-culture adaptation reveals extensive changes over time

**DOI:** 10.1038/s41598-017-03318-3

**Published:** 2017-06-13

**Authors:** Haiyan Wei, Jonathan Audet, Gary Wong, Shihua He, Xueyong Huang, Todd Cutts, Steven Theriault, Bianli Xu, Gary Kobinger, Xiangguo Qiu

**Affiliations:** 1 Institute of Infectious Disease, Henan Center for Disease Control, Henan, China; 2 Special Pathogens Program, National Microbiology Laboratory, Public Health Agency of Canada, Winnipeg, Manitoba Canada; 3 Department of Medical Microbiology, University of Manitoba, Winnipeg, Canada; 4 CAS Key Laboratory of Pathogenic Microbiology and Immunology, Institute of Microbiology, Chinese Academy of Sciences, Beijing, China; 5 Applied Biosafety Research Program, National Microbiology Laboratory, Public Health Agency of Canada, Winnipeg, Manitoba Canada; 6 Department of Immunology, University of Manitoba, Winnipeg, Canada; 7 Department of Pathology and Laboratory Medicine, University of Pennsylvania School of Medicine, Philadelphia, PA USA; 8 Centre de Recherche en Infectiologie, Centre Hospitalier Universitaire de Québec, Université Laval, Québec City, Québec Canada

## Abstract

Marburg virus (MARV) has caused outbreaks of filoviral hemorrhagic fever since its discovery in 1967. The largest and deadliest outbreak occurred in Angola in 2005, with 252 cases and 227 deaths. In 2014, we developed a mouse-adapted MARV, Angola variant through serial passaging in mice. The mouse-adapted MARV exhibits many of the hallmarks of MARV disease in humans. By applying deep-sequencing to every passage of the virus, we are able to study virus evolution in this host with surprising precision. We show that two regions go through substantial changes: the intergenic region between NP and VP35, as well as the first 100 amino acids of the VP40 protein. Our results also reveal that there were profound changes during the production of the final virus stock in cell culture. Overall, our results show that a handful of regions carry most of the mutations acquired during the adaptation of the virus to a new host and that many mutations become fixed very early during the adaptation process.

## Introduction

Marburg virus (MARV) belongs to the family *Filoviridae* as a member of the species *Marburg marburgvirus* ^1^. It has been causing sporadic outbreaks in Central Africa since at least 1975^2^. MARV causes a hemorrhagic fever characteristic of the filoviruses with a case-fatality rate in humans ranging from ~20% to 90%^3, 4^. The virus is found in fruit bats and transmitted to humans as a zoonotic disease^5^. The genus *Marburgvirus* includes a single species (*Marburg marburgvirus*) which includes two viruses: Marburg virus and Ravn virus (RAVV). Live replication-competent viruses have been isolated from fruit bats caught in Africa^5^. Recent studies have shown that both viruses and their variants can be found circulating within the same bat colonies^5^.

While MARV can cause outbreaks as lethal as Ebola virus, countermeasures against MARV, such as vaccines and treatments, are much less developed. This was due, in large part, to the absence of good small animal-adapted viruses. The lack of such viruses meant that testing of countermeasures had to be performed in animals that were at least partially immunosuppressed or in non-human primates. In 2014, we developed a mouse-adapted MARV based on the Angola variant (MARV-Angola)^6^. This variant was chosen because it caused the largest and deadliest recorded MARV outbreak.

The adaptation of the virus required 24 passages in SCID mice. At that point, the virus was shown to be equally lethal to C57BL/6 and BALB/c mice. The disease caused by the adapted virus mimicked the liver damage and coagulation abnormalities observed in human cases. The virus was adapted by infecting new mice with liver homogenates collected on day 7 post-infection. Samples of the liver homogenates were retained. These samples were used to recover viral RNA from 23 of the 24 passages.

The samples retained from the adaptation allow us to use deep-sequencing to look at the adaptation process at a level of detail that was previously not possible with past sequencing technologies. We believe that understanding the sequence and frequencies of mutations during adaptation will allow better monitoring of MARV and the related ebolaviruses for changes that may indicate adaptation to a new host and potentially identify locations in the viral genome that are associated with virulence.

The most active adaptation was found within the VP40 gene as well as the NP-VP35 intergenic region. We also sequenced a virus stock after 2 passages in Vero E6 cells. This stock lost a large number of mutations seen in the last mouse passage; however, the MARV-Angola stock retained high levels of lethality to mice. This result points to a handful of mutations as being crucial for causing MARV disease in mice.

## Results

### Amplification and coverage

**Figure 1:**
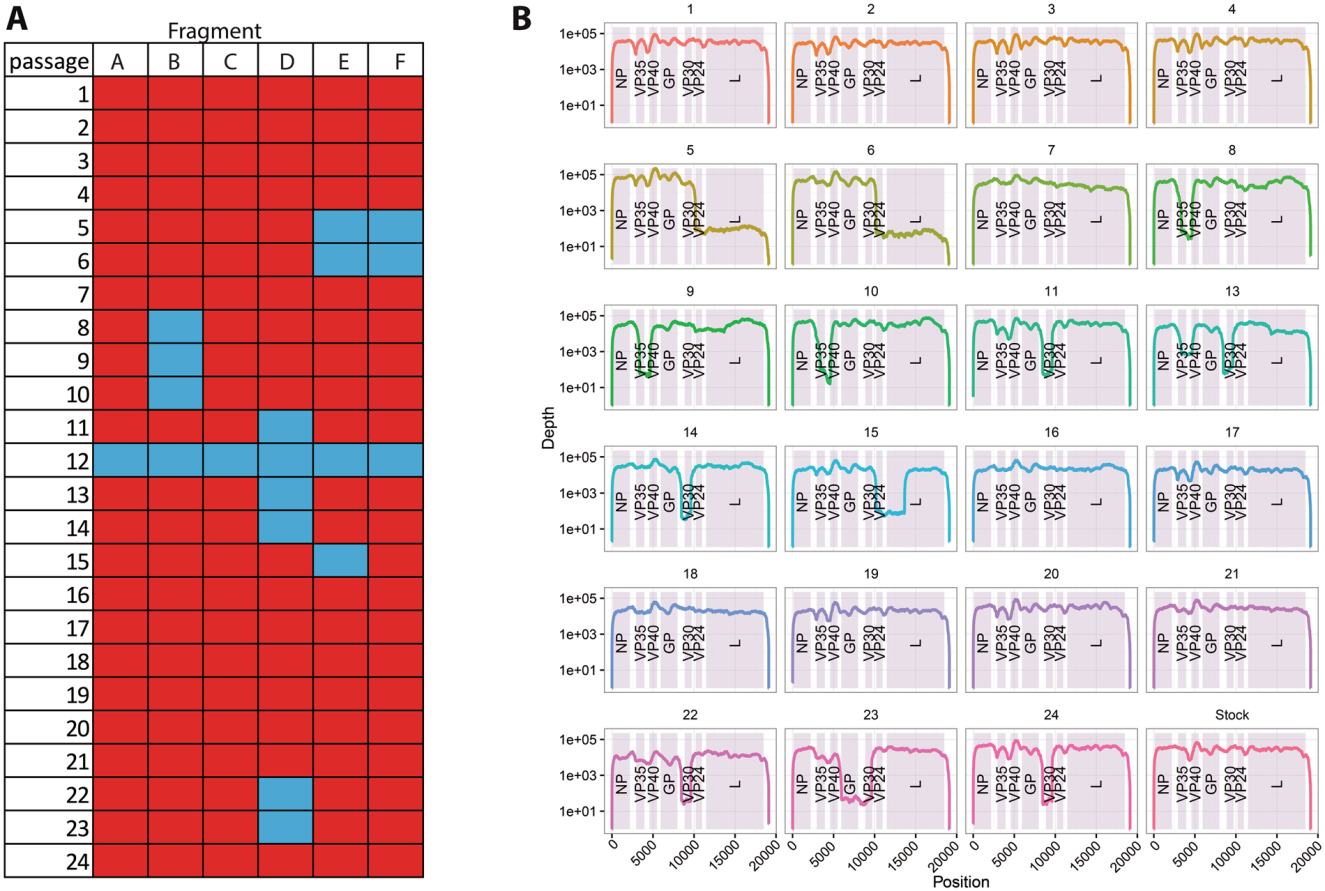
Amplification and sequencing coverage. (**A**) Summary of the PCR amplification. Red squares are fragments that were amplified, blue squares represent fragments that could not be amplified. For each passage (line), 6 overlapping fragments were amplified (column) by RT-PCR. (**B**) MiSeq sequencing coverage. Passage 25 is the Virus Stock. Coding regions are highlighted.

We performed deep-sequencing on liver homogenate samples from each passage during the adaptation of MARV to mice. All passages except for passage 12 were amplified fully or partially by RT-PCR (Fig. 1a). The median coverage was 25 742 fold (IQR: 19 718; range: 0–223 692) (Fig. 1b). The regions which were known not to have been amplified by PCR still had coverage of < 200 fold, for that reason the minimum coverage used in variant calling was set to 200 fold.

### Adaptation of MARV to mice

**Figure 2:**
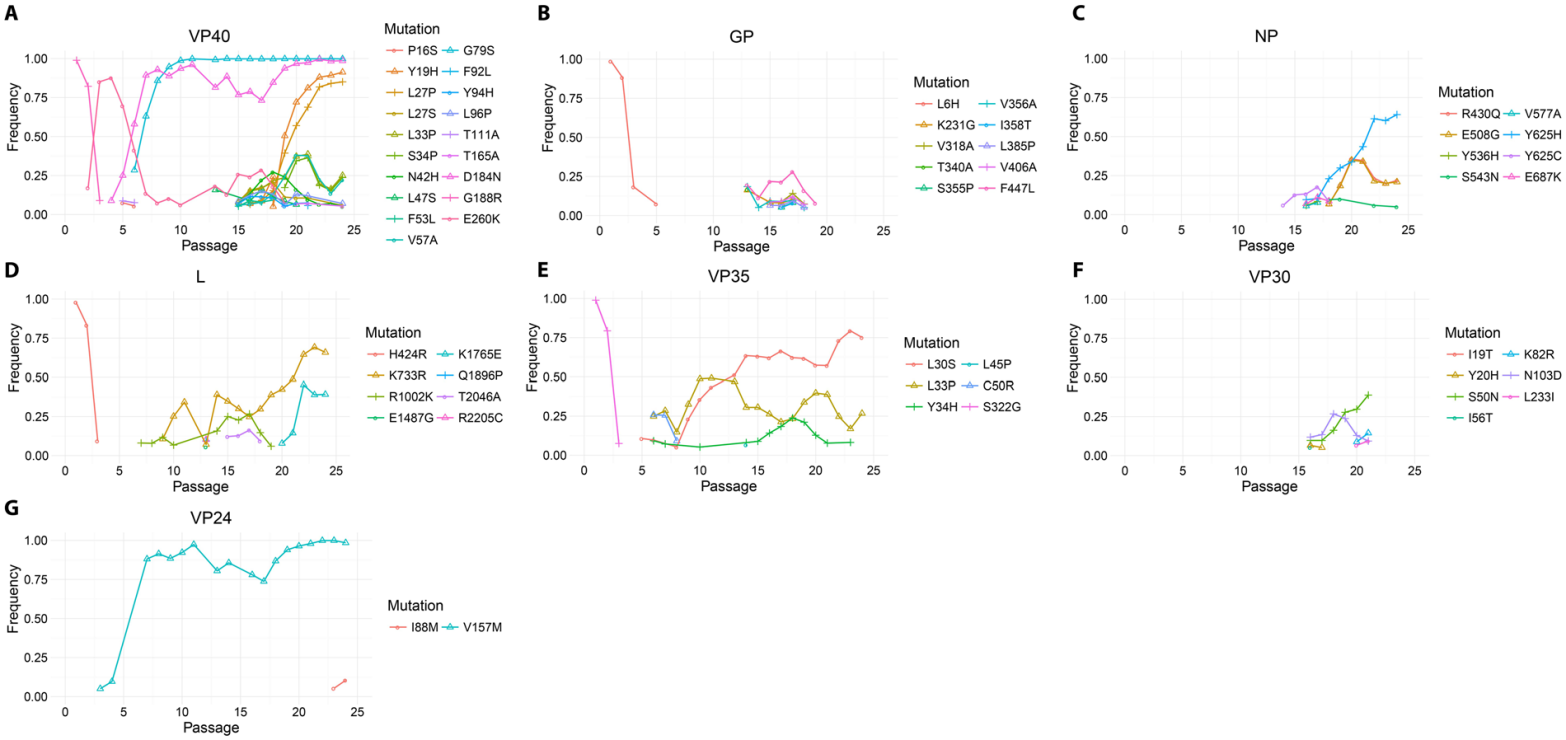
Non-silent mutations in coding regions over virus passages in SCID mice. Frequency of reads containing a mutation over depth as a function of passage for: (**A**) VP40; (**B**) GP; (**C**) NP; (**D**) L; (**E**) VP35; (**F**) VP30; and (**G**) VP24. For each protein, the mutations are colored from the most N-terminal (Red) to the most C-terminal (Pink).

All MARV proteins went through some degree of mutations (Fig. 2a–g). A total of 54 silent mutations were found during the adaptation (Supplementary Figure 1). Only 7 of those silent mutations remained in the virus stock after two passages in cell culture (Supplementary Table 1). However, the VP40 protein was where most of the changes happened with 19 mutations appearing over the adaptation period, specifically in the first 100 N-terminal amino acids (14 mutations; Supplementary Figure 2). The changes in VP40 started very early during the adaptation process. The glycoprotein (GP) also had a large number of mutations (10 mutations; Supplementary Figure 3), most of which were located in the mucin-like domain. However, none of the mutations in the GP became dominant in the virus population and the mutations were only detected between passages 13 and 19. The NP and L protein each had 8 mutations, and only one of those for each protein was shown to be dominant in the population by passage 24. The L protein started to accumulate mutations around the 5^th^ passage. The NP, on the other hand, started to adapt only after 13 passages. We detected 6 mutations in the VP35, 5 of them in the first 50 N-terminal amino acids. The VP35 started mutating at passage 5. We also detected 6 mutations in VP30, 4 of them in the first 100 N-terminal amino acids and all 6 mutations were only detected during passages 16 to 21. The VP24 only went through 2 mutations during the adaptation process. Interestingly, one mutation (V157M) appeared after the 4^th^ passage and became dominant starting at passage 7. Unfortunately this fragment could not be amplified for passages 5 and 6.

**Figure 3:**
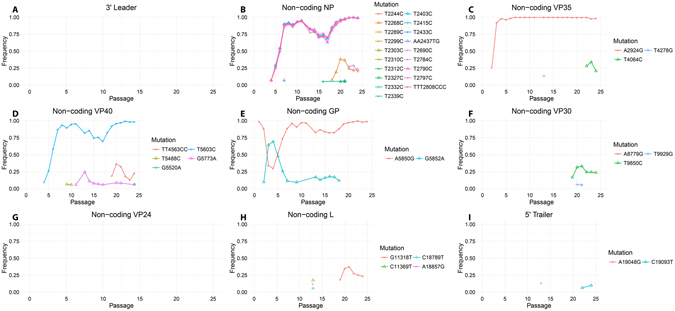
Mutations in non-coding regions over virus passages in SCID mice. Frequency of reads containing a mutation over depth as a function of passage for the following non-coding regions: (**A**) 3′ leader; (**B**) NP; (**C**) VP35; (**D**) VP40; (**E**) GP; (**F**) VP30; (**G**) VP24; (**H**) L; and (**I**) 5′ trailer. For each protein, the mutations are colored from the most 3′ (Red) to the most 5′ (Pink).

No mutations were detected in the intergenic regions. However, the non-coding regions did undergo mutations during the adaptation (Fig. 3a–i). Most non-coding regions saw 4 mutations or fewer, with the 3′ leader and the non-coding regions of VP24 not changing from the initial stock at all. When mutations were detected, many of them were only present for one or a few passages and did not reach high percentages of the population. The 5′ end of the non-coding region of NP did see a large number of mutations. This region underwent 19 mutations, many of which were in clusters, becoming fixed by passage 24 (Supplementary Figure 4). Outside of this region, only 3 mutations became fixed or nearly fixed, one each in the non-coding regions of VP35, VP40, and GP.

### Effect of cell culture passaging

**Figure 4:**
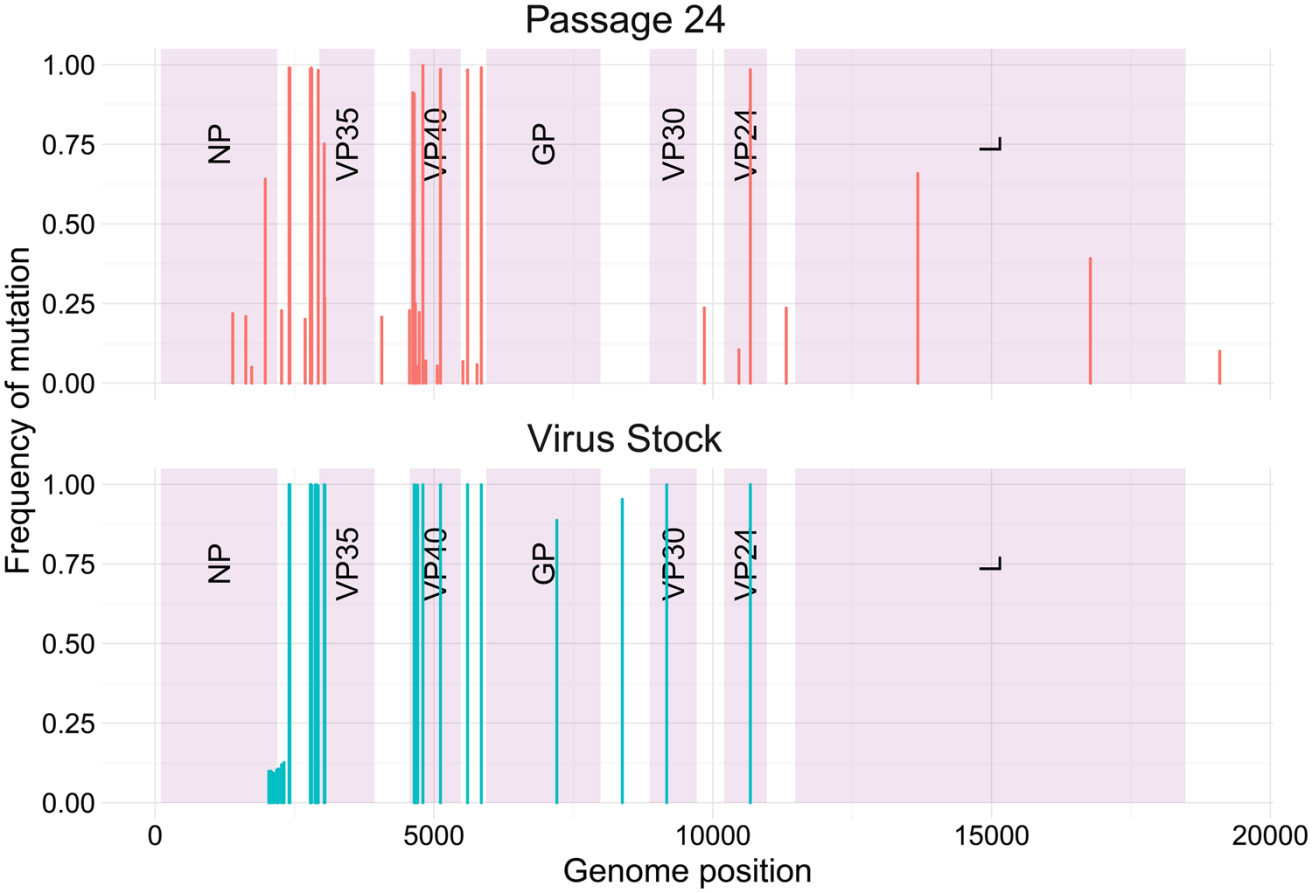
Location and frequency of each mutation at Passage 24 and in the Virus Stock. Each vertical bar represents one mutation.

In order to understand the effect of cell culture passaging, we also sequenced the virus stock used to characterize the model. The virus stock was produced by passaging the passage 24 isolate twice in Vero E6 cells to produce a large virus stock that can be used for subsequent studies. A large number of mutations from the passage 24 isolate were lost during the stock production, but a few new mutations appeared (Fig. 4, Table 1). These new mutations were concentrated in the C-terminal and non-coding region of NP (genome position 2039–2267) and the non-coding region of VP35 (genome position 2870–2915). Most mutations with frequencies below 25% at passage 24 were lost, except for 2 mutations in VP40 (L27S, and N42H) which jumped from 5% of the population at passage 24 to 100% of the population in the stock. The mutations which appeared after Vero cell passaging at the C-terminus of NP and the subsequent non-coding regions (2039–2310) had very low frequencies (~10%) and included a mutation to the stop codon. The mutations which appeared after Vero cell passaging in the non-coding region of VP35 were all fixed (frequency of 1). The mutations present at passage 24 were either lost entirely or fixed. The mutations which were acquired during cell passaging (i.e. not detected at passage 24) were present in the virus stock at either lower (~9–13%) or very high frequencies (89–100%). All of the mutations previously identified in Sanger sequencing of the Vero-passaged virus stock^6^ were also found, except for mutations at positions 19104 and 19105. The coverage of the final stock dropped below the 200 minimum by position 19094, therefore no variants were called beyond that position.

**Table 1:** Comparison of mutations present in passage 24 and in the virus stock.

Genomic position^@^	Region	Coding (c), non-coding (nc), or intergenic (ig) region	Passage 24 frequency*	Virus Stock frequency*	AA mutation	Nucleic Acid mutation^#^
1392	NP	c	0.22	—	R430Q	G1392A
1626	NP	c	0.21	—	E508G	A1626G
1731	NP	c	0.05	—	S543N	G1731A
1975	NP	c	0.64	—	Y625H	T1975C
1976						T1976C
2039	NP	c	—	0.1	Y646H	T2039C
2079	NP	c	—	0.1	L659P	T2079C
2080						T2080C
2102	NP	c	—	0.09	Y667H	T2102C
2130	NP	c	—	0.09	L676P	T2130C
2181	NP	c	—	0.1	M693T	T2181C
2189	NP	c	—	0.09	STP696Q	T2189C
2211	NP	nc	—	0.11		T2211C
2265	NP	nc	—	0.12		T2265C
2267	NP	nc	—	0.12		T2267C
2268	NP	nc	0.23	—		T2268C
2310	NP	nc	—	0.13		T2310C
2403	NP	nc	0.99	1		T2403C
2415	NP	nc	0.99	1		T2415C
2690	NP	nc	0.2	—		T2690C
2784	NP	nc	0.99	1		T2784C
2790	NP	nc	0.99	1		T2790C
2797	NP	nc	0.99	1		T2797C
2808	NP	nc	0.98	1		T2808C
2809						T2809C
2810						T2810C
2870	VP35	nc	—	1		T2870C
2871						T2871C
2874	VP35	nc	—	1		T2874C
2876	VP35	nc	—	1		T2876C
2879	VP35	nc	—	1		T2879C
2892	VP35	nc	—	1		T2892C
2897	VP35	nc	—	1		T2897C
2899	VP35	nc	—	1		T2899C
2902	VP35	nc	—	1		T2902C
2904	VP35	nc	—	1		T2904C
2907	VP35	nc	—	1		T2907C
2908						T2908C
2911	VP35	nc	—	1		T2911C
2912						T2912C
2913						T2913C
2914						T2914C
2915						T2915C
2924	VP35	nc	0.98	—		A2924G
2924	VP35	nc	—	0.99		A2924G
2925						T2925C
2926						T2926C
3033	VP35	c	0.75	1	L30S	T3033C
3042	VP35	c	0.27	—	L33P	T3042C
3044	VP35	c	—	1	Y34H	T3044C
4064	VP35	nc	0.21	—		T4064C
4563	VP40	nc	0.23	—		T4563C
4564						T4564C
4621	VP40	c	0.91	—	Y19H	T4621C
4622						T4622C
4646	VP40	c	0.85	—	L27P	T4646C
4647						T4647C
4646	VP40	c	0.06	1	L27S	T4646T
4647						T4647C
4665	VP40	c	0.25	0.97	L33P	T4665C
4667	VP40	c	0.24	—	S34P	T4667C
4691	VP40	c	0.05	1	N42H	A4691C
4707	VP40	c	—	1	L47S	T4707C
4737	VP40	c	0.22	—	V57A	T4737C
4802	VP40	c	1	1	G79S	G4802A
4853	VP40	c	0.07	—	L96P	T4853C
4854						T4854C
5060	VP40	c	0.05	—	T165A	A5060G
5117	VP40	c	0.99	1	D184N	G5117A
5520	VP40	nc	0.07	—		G5520A
5603	VP40	nc	0.98	1		T5603C
5773	VP40	nc	0.06	—		G5773A
5850	GP	nc	0.99	1		A5850G
7204	GP	c	—	0.89	N422D	A7204G
8378	GP	nc	—	0.95		C8378T
9175	VP30	c	—	1	N103D	A9175G
9850	VP30	nc	0.24	—		T9850C
10470	VP24	c	0.1	—	I88M	A10470G
10675	VP24	c	0.98	1	V157M	G10675A
11318	L	nc	0.24	—		G11318T
13678	L	c	0.66	—	K733R	A13678G
16773	L	c	0.39	—	K1765E	A16773G
19093	5′ Trailer	ig	0.1	—		C19093T

^@^Multiple sites in the same cell were found to always move together.

* “—” represents mutations that were not detected.

^#^Based on positive-sense sequence.

## Discussion

We previously published a new MARV-Angola model, in which the virus was adapted to produce lethal infection in adult immunocompetent mice. Samples from each of the 24 passages in mice were preserved, unfortunately not in sufficient quantities to determine the viral load accurately. Therefore the titer of each sample may be different (lower titers in the early passages but reaching titers of 10^10^ TCID_50_/gram tissue in the last passages^6^), which has an impact on the rate of virus mutation, as larger viral population sizes result in stronger clonal interference and hence more mutations competing for fixation^7^. It is possible that different mutations may arise and become fixed under different conditions (ie. substantial changes in virus titer). Nevertheless, we were still able to use this rare opportunity to study the changes in the virus population after each passage.

We have observed a large number of mutations in two main regions: the VP40 protein and the NP-VP35 intergenic region. Although the VP40 underwent the largest number of mutations, all the proteins did go through some mutations. The VP30 and GP, however, only went through transient periods of mutations before reverting to the wild-type sequence before passage 24.

The extent of mutations in VP40 was interesting but not unexpected. One of the main functions of VP40 in MARV immunopathology is to block the host cell responses to interferon signalling^8^. As such, the VP40 needs to interact with multiple host proteins which will be very different between mice and primates. Many of the mutations are located in the N-terminal domain (amino acids 38–138) which is required and sufficient for the blockade of interferon signalling^9^. One interesting mutation in this region is G79S, which appears at passage 6 and becomes essentially fixed by passage 10. This mutation was found to confer interferon antagonism in mouse cells *in vitro* ^10^. Another mutation in VP40, D184N, is interesting because it was found to provide MARV Musoke VP40 with a replicative advantage in guinea pig cells^11, 12^. Also, its frequency very closely matches that of the V157M mutation in VP24.The same temporal pattern is also followed by 6 mutations in the non-coding regions of NP (T2415C, T2433C, T2784C, T2790C, T2797C, and the [T2808C, T2809C, T2810C] group) and by 1 mutation in the non-coding region of VP40. Finally, the VP40 mutation E260K also presents interesting patterns. It appears earlier than the D184N mutation and almost reaches fixation, until the appearance of D184N, at which point the virus population appears to be split between the two mutations (the sum of their frequencies is approximately equal to 1). The frequency of viruses carrying E260K always moves in the opposite direction from D184N. Thus, these mutations appear to be mutually exclusive.

While the VP35 is also involved in immune evasion, its main role is to interact with the viral dsRNA to prevent its detection by the host cells^13, 14^. Thus, VP35 does not interact with host proteins as much as VP40. Surprisingly, no mutations were left in the glycoprotein by the final passage, suggesting there was no need to adapt to the host cell surface receptors. Supporting that hypothesis is the fact that the mutations were almost all found within the mucin domain, which is cleaved before receptor interaction in the endosome. The adaptation took place in SCID mice, which are unable to mount an adaptive immune response, T and B cells responding to a specific pathogen. This is another possible reason for the lack of major changes in the GP. Its mucin domain contains many targets for antibodies and T cell responses; this domain is known to be highly disordered and prone to mutations, which allow the virus to escape adaptive responses. In the absence of a normal immune response, there was no selection pressure on the GP; this absence of adaptation did not seem to affect virulence, as the virus was also fully lethal to wild-type mice. The L, NP, and VP30 also had a number of mutations which accumulated by the end of the passaging. While these proteins mainly function together as the transcription/replication unit, they also likely interact with certain host proteins, although most of those proteins remain ill-defined.

The mutations found in the coding regions are consistent with those reported during the adaptation of MARV-Ci67^15^; although we were able to detect mutations in more coding regions. The VP40 mutation D184N appeared in their 3^rd^ passage (4^th^ passage in this experiment) along with G79S shortly before the virus became fully lethal to SCID mice. We also detected the presence of VP40 mutation Y19H but not I187T (we did find G188R) or I228M and we detected NP mutation Y536H but not GP mutation F445P. The two mutations found to restore the anti-interferon activity of RAVV VP40 in mouse cells (V57A and T165A) appeared during the adaptation but were absent from the final stock. Since this stock was found to be lethal to immunocompetent mice, other mutations may be compensating for the effect of V57A and T165A. Interestingly, even though Lofts *et al*. passaged their virus repeatedly in immunocompetent animals only one mutation appeared in the GP gene, suggesting that the MARV GP does not need adaptation between primate- and mouse-adapted viruses.

The mutations in the non-coding regions of NP were extensive and somewhat unexpected. While we were expecting that the non-coding and/or intergenic regions would mutate to properly modulate protein expression for the new host, the number of mutations was much higher than that of any other non-coding region. The two genes located downstream of this region, VP35 and VP40, are both very important for immune evasion, their expression very likely needs to be re-modulated to fit the pressures on the virus in a new host. A similar change has been noted in sequences obtained during the Ebola virus disease outbreak in West Africa, in which a recent study has uncovered that mutations in two sites in the equivalent non-coding region of Ebola virus lead to increased virulence^16^. Based on minigenome experiments, it was shown that these mutations could alter the expression of genes both upstream and downstream.

We also looked into the effect of cell culture passaging. We observed two main overarching effects. First, many mutations still present in most of the virus population after passage 24 disappeared after production of the virus stock. Second, the few mutations which remained after passaging the virus in cell culture were mostly fixed in the virus population. These two results suggest that even without plaque purification, cell culture passaging of MARV leads to selection and mutation of the virus. While our results cannot confirm that the changes are adaptive, it is interesting to note that the cells being infected are from NHPs, requiring a degree of balance between mouse-adapted and “cell-adapted” viruses. Many of the new mutations appeared in the NP-VP35 intergenic region, suggesting that adaptation to cell culture affects gene regulation rather than protein sequences.

Overall, our results offer a detailed glimpse into the changes which occur during virus adaptation to a new host and the first study of this type for a filovirus. In turn, these results show “hot-spots” for adaptation and monitoring of adaptation, these include: the 5′ NP non-coding region, the first 100 amino acids of the VP40 protein, and the VP35 protein.

## Methods

### Biosafety

All experiments involving live, replication-competent Marburg virus were carried out inside the Bio-Safety Level 4 laboratory at the National Microbiology Laboratory of the Public Health Agency of Canada in the Canadian Science Center for Human and Animal Health.

### RNA extraction

The RNA was extracted from 140 µl of liver homogenate supernatant using a Qiagen Viral RNA Mini kit, according to the manufacturer’s instructions. Elution was performed in 60 µl.

### RT-PCR

The reverse transcription was carried out using the SuperScript III RT enzyme (ThermoFisher) following the manufacturer’s instructions. Four microliters of RNA were used in the reaction along with 250 ng of random hexamers (ThermoFisher). The subsequent PCR reactions were carried out using Phusion Green Hi-Fidelity PCR mix (ThermoFisher) in 50 µl, using 2 µl of the RT product as template, primers at 200 nM, and 1 U of Phusion enzyme. The PCR conditions were 35 cycles of: 98 °C 30 s, 55 °C 30 s, 72 °C 3 min; and a final extension at 72 °C for 10 min. The genome was amplified in 6 overlapping fragments of 3–6 kb (named A through F). In some cases, some fragments could not be recovered; for a few passages where fragment F could not be amplified, two subfragments F1 and F2 were successfully amplified. In all other cases where fragments were not amplified by the primers in Supplementary Table 2, alternate primers were used, without success. The reactions were run on a 1% agarose gel and bands of the expected size were cut out and purified using a Qiagen Gel Extraction kit according to the manufacturer’s instructions.

### Library construction and MiSeq sequencing

The amplicons were quantitated using the PicoGreen dye and fragments from the same passage were pooled at equimolar concentration using a BioMek FX (BioTek). Library construction was done using the Nextera DNA Sample preparation kit (24-Sample) (Illumina) as per manufacturer’s instructions. Sequencing was carried out using a MiSeq sequencer (Illumina) using the MiSeq Reagent Kit v3 (600 cycles) (Illumina).

### Alignment of the reads and variant calling

Paired reads from each passage were aligned to a reference sequence (GenBank accession DQ447660.1) using Bowtie 2 version 2.0.5. Conversion to bam files, sorting, and indexing were performed using Samtools version 0.1.18. Variant calling was performed using freebayes version 0.9.8 using a ploidy of 4, a max complex gap of 0, a minimum quality score of 30, a minimum frequency of 5%, a minimum coverage of 200 fold, and to ignore insertions and deletions. The frequencies of variants were determined as the number of reads with a specific variant over the depth at that location. Finally, coverage was determined using bedtools version 2.25.0.

### Data availability and analysis

All assembled files (*.bam) are available on the Sequence Read Archive (SRA) under the BioProject accession number PRJNA320428. The coverage results files and variant calls, as well as the scripts used to produce the figures are available on the Open Science Framework, at http://osf.io/8sprb (DOI: 10.17605/OSF.IO/8SPRB).

## Electronic supplementary material

Supplementary Material
