# Supplementary material for: Deep-sequencing of Marburg virus genome during sequential mouse passaging and cell-culture adaptation reveals extensive changes over time
Source: Sci Rep. 2017 Jun 13;7:3390. doi: 10.1038/s41598-017-03318-3 (PMC5469859; doi:10.1038/s41598-017-03318-3)
Supplement: Supplementary file 1 — Supplementary Material [file 41598_2017_3318_MOESM1_ESM.pdf]

Deep-sequencing of Marburg virus genome during sequential mouse passaging and cell-culture adaptation reveals extensive changes over time

Haiyan Wei[1,2]*, Jonathan Audet[3]*, Gary Wong[2,4], Shihua He[2], Xueyong Huang[1], Todd Cutts[5], Steven Theriault[5], Bianli Xu[1], Gary Kobinger[2,3,6,7,8], Xiangguo Qiu[2,3#]

Supplementary Figure 1

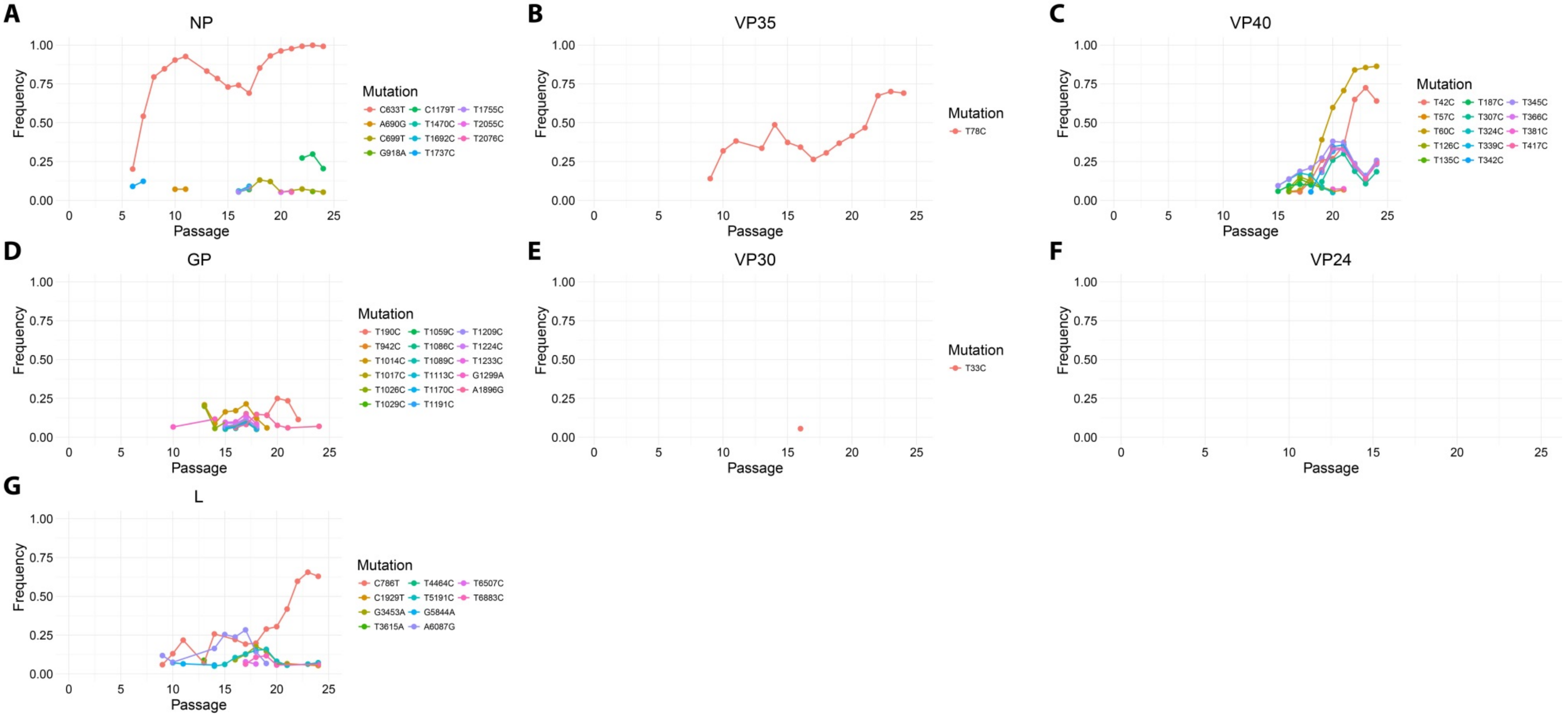

Silent mutations in coding regions over virus passages in SCID mice. Proportion of reads containing a mutation over depth as a function of passage for: A) VP40; B) GP; C) NP; D) L; E) VP35; F) VP30; and G) VP24. For each protein, the mutations are colored from the most N-terminal (Red) to the most C-terminal (Pink).

Supplementary Figure 2

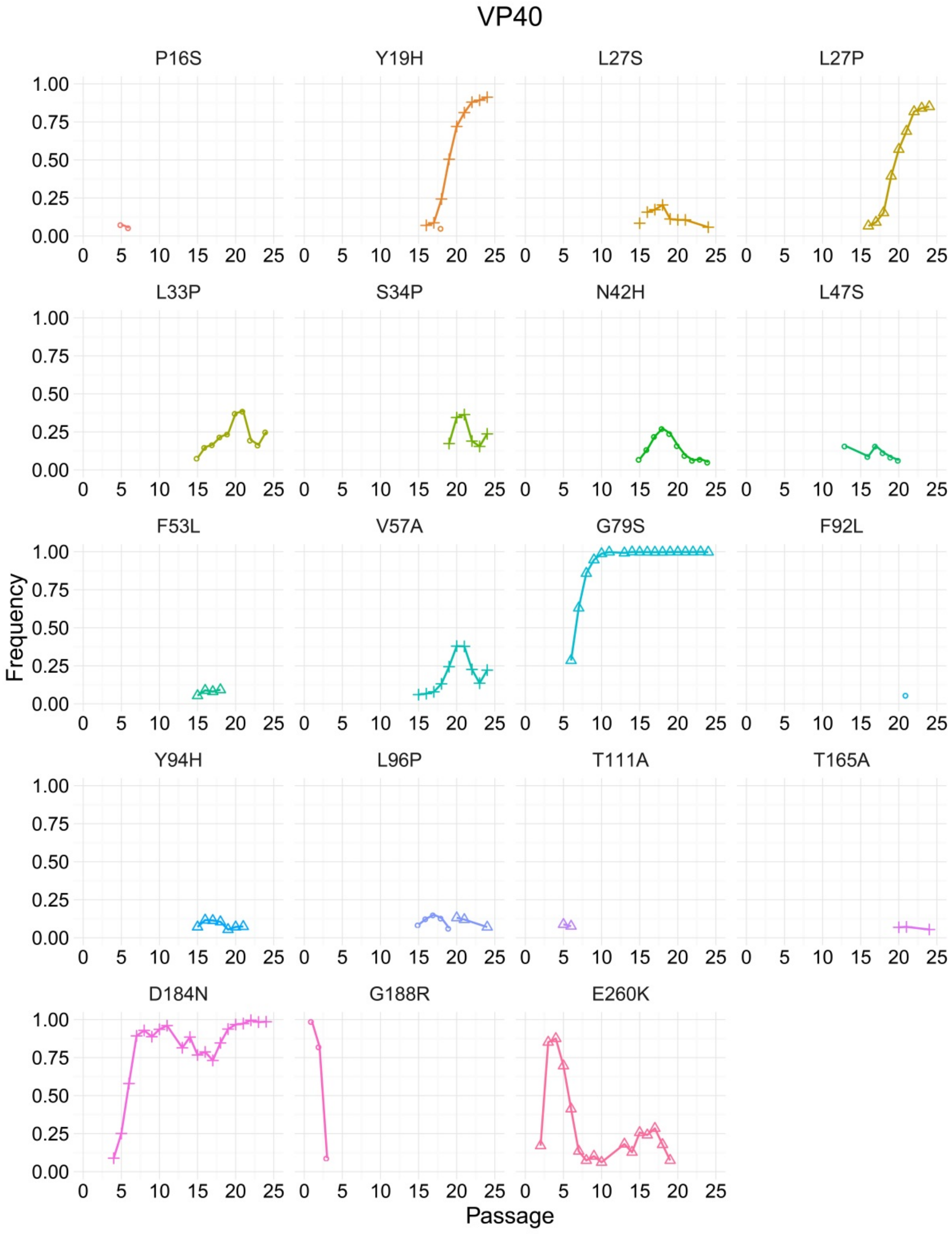

Individual non-silent mutations in VP40. The same data as in Figure 2A, but each mutation is plotted in its own graph, based on the amino acid substitution.

Supplementary Figure 3

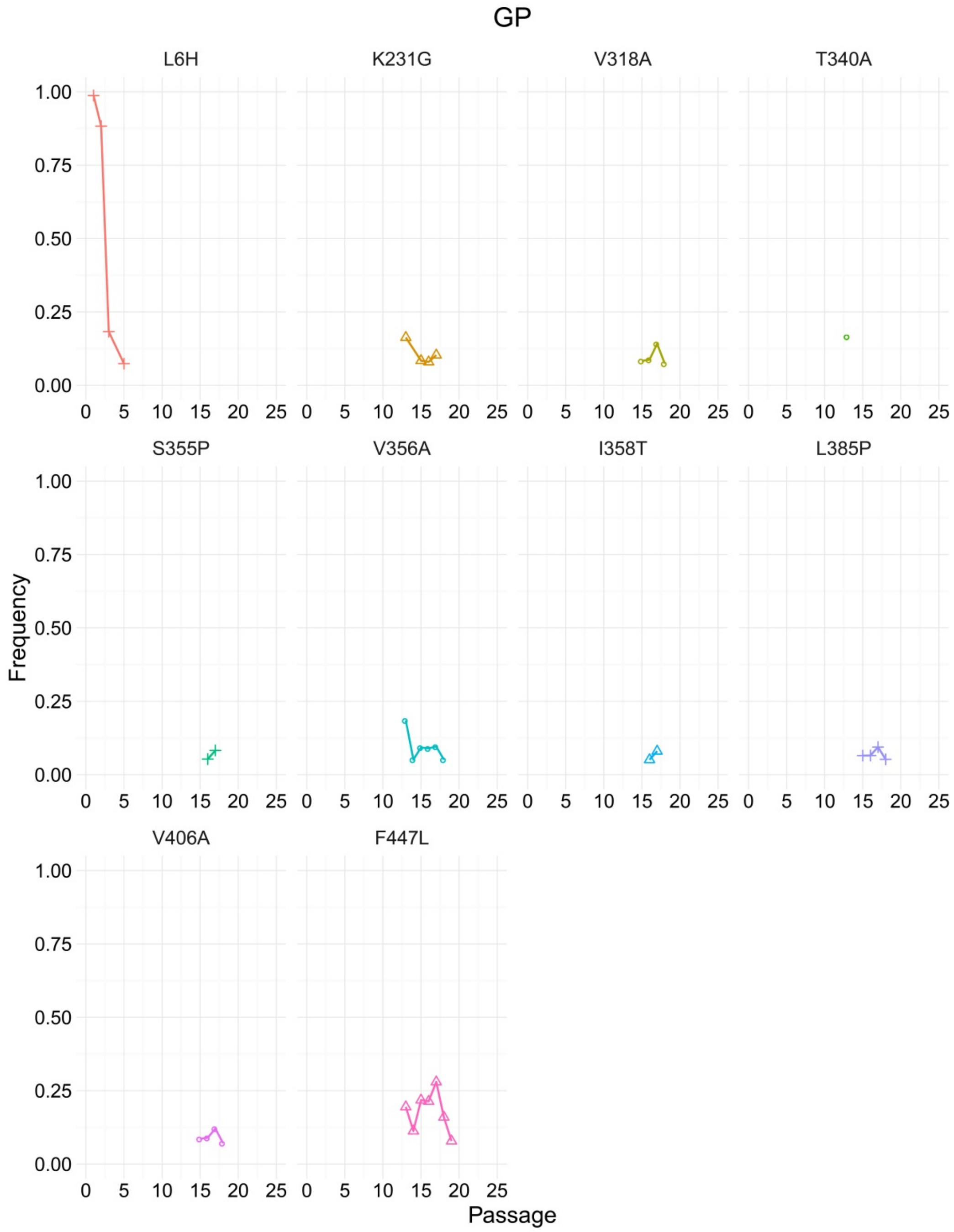

Individual non-silent mutations in GP. The same data as in Figure 2B, but each mutation is plotted in its own graph, based on the amino acid substitution.

Supplementary Figure 4

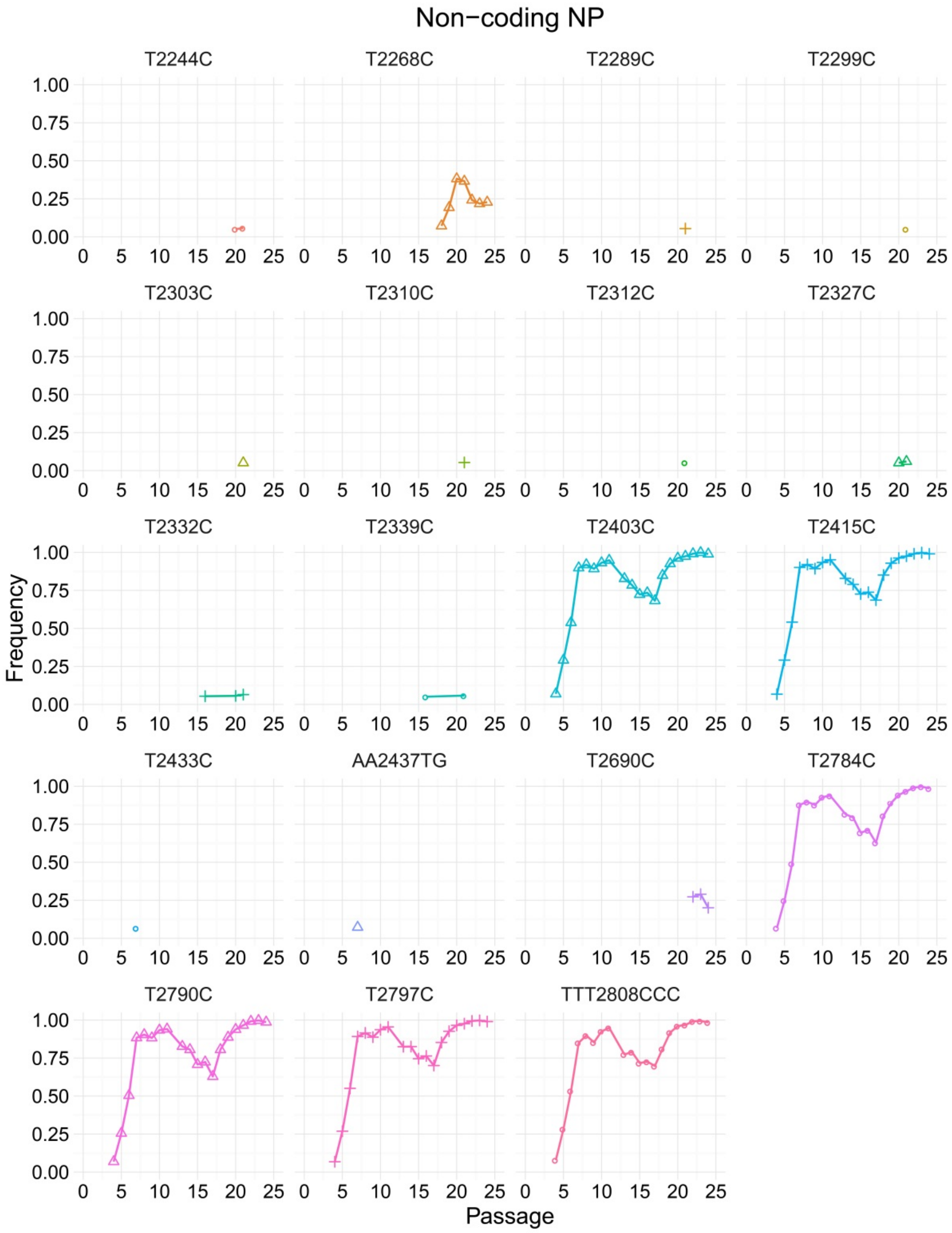

Individual mutations in the NP-VP35 intergenic region. The same data as in Figure 3B, but each mutation is plotted in its own graph.

Supplementary Table 1: Changes in frequencies of silent mutations between the final passage and the virus stock

Protein	Genome Position	Frequency		Mutation*
		Passage 24	Virus Stock	
NP	736	0.99	1	C633T
NP	802	0.05	0	C699T
NP	1282	0.2	0	C1179T
NP	2044	0	0.09	T1941C
NP	2068	0	0.09	T1965C
VP35	2962	0	1	T18C
VP35	2984	0	1	T40C
VP35	3022	0.69	0	T78C
VP40	4609	0.64	0	T42C
VP40	4627	0.86	0	T60C
VP40	4874	0.18	0	T307C
VP40	4906	0.23	0	T339C
VP40	4909	0.25	0	T342C
VP40	4912	0.26	0	T345C
VP40	4933	0.24	0	T366C
VP40	4984	0.24	0	T417C
GP	7836	0.07	0	A1896G
L	12266	0.63	0	C786T
L	12398	0	0.1	T918C
L	13409	0.05	0	C1929T
L	14933	0	1	G3453A
L	16671	0.07	0	T5191C
L	18363	0.06	0	T6883C

*Numbered from the start of the appropriate coding region.

Supplementary Table 2. Primers used in whole genome amplification of MARV/Ang-MA.

Primer Name	Fragment	Sequence	Direction	Start*	Stop*
MARV-1F	A	AGACACACAAAAACAAGAGATG	F	1	22
MARV-3.5F	B	GTTCTTCCAGTTGAGATTAC	F	686	705
MARV-8F	C	GGCGGATCAGCTATCAAATC	F	4654	4673
MARV-11F	D	TCAAGCTCACTAGCACCTCAA	F	6665	6685
MARV-16F	E	ATATGTGATGAACAATATCC	F	9618	9637
MARV-22F	F, F1	GTATATCATGTGCCCAAATC	F	13642	13661
MARV-25F	F2	GGTCAAGCTGCGATTGATAG	F	15471	15490
MARV-57R	A	GTTCTTCCAGTTGAGATTAC	R	3366	3347
MARV-52.5R	B	GATAAGGAGACATGTGG	R	5964	5948
MARV-49R	C	GAAGTAAGGCAAGTTGTT	R	8475	8458
MARV-46R	D	ATCCTATACTGCAAAGCAAT	R	10180	10161
MARV-40R	E	GTGAGAGGATCACTTATGTT	R	14278	14259
MARV-37R	F1	GTGGATGATATAAGGAATGT	R	16048	16029
MARV-32R	F, F2	TGGACACACTAAAAAGATGA	R	19114	19095

*Start and Stop positions are based on the GenBank sequence DQ447660.1
